# Overexpression of an Antisense RNA of Maize Receptor-Like Kinase Gene *ZmRLK7* Enlarges the Organ and Seed Size of Transgenic *Arabidopsis* Plants

**DOI:** 10.3389/fpls.2020.579120

**Published:** 2020-11-11

**Authors:** Chunmei He, Juan Wang, Rui Dong, Haiying Guan, Tieshan Liu, Chunxiao Liu, Qiang Liu, Liming Wang

**Affiliations:** Maize Research Institute, Shandong Academy of Agricultural Sciences/National Engineering Laboratory of Wheat and Maize/Key Laboratory of Biology and Genetic Improvement of Maize in Northern Yellow-huai River Plain, Ministry of Agriculture, Jinan, China

**Keywords:** *ZmRLK7*, LRR-receptor like kinase, maize, overexpression, *Arabidopsis*, organ and seed size

## Abstract

Leucine-rich repeat (LRR)-receptor-like protein kinases (LRR-RLKs) play vital roles in plant growth, development, and responses to environmental stresses. In this study, a new LRR-RLK gene, *ZmRLK7*, was isolated from maize, and its function within plant development was investigated through ectopic expression in *Arabidopsis*. The spatial expression pattern analysis reveals that *ZmRLK7* is highly expressed in embryos prior to programmed cell death (PCD) of starchy endosperm tissues, and its encoded protein has been localized to both plasm and nuclear membranes subcellularly. Overexpression of sense *ZmRLK7* reduced the plant height, organ size (e.g., petals, silique, and seeds), and 1000-seed weight in transgenic lines, while the antisense transgene enlarged these traits. Cytological analysis suggested that ZmRLK7 negatively regulates petal size through restricting both cell expansion and proliferation. In addition, abnormal epidermal cell structure was observed, and the stomata number decreased obviously in sense *ZmRLK7* transgenic lines with a lower stomatal index than that in the wild type. Quantitative RT-PCR analysis indicated that transcript levels of genes that are involved in the brassinosteroid and ERACTA signaling pathways were coordinately altered, which could partially explain the phenotypic variation. Moreover, overexpression of antisense *ZmRLK7* substantially rescued the *Arabidopsis bak1-3* mutant phenotype. All these results together suggest that *ZmRLK7* can serve as an important regulator in regulating plant architecture and organ size formation. This work will provide insight into the function of *ZmRLK7* in maize.

## Introduction

In plant growth and development, the organ size and shape of a plant are complicated biological features that are mainly determined by coordination of cell proliferation, expansion, and differentiation (Mizukami, 2001). Classical plant hormones have been considered as the main regulators of plant morphogenesis, however, cell–cell communication mediated by transmembrane receptor-like kinases (RLKs) has been revealed to be a clear and vital part of plant development in recent years. Leucine-rich repeat (LRR)-RLKs are the largest class of transmembrane receptor protein kinases in plants, and they typically contain an extracellular receptor domain (LRR) with which to perceive the specific signal, a transmembrane domain (TM) within the membrane, and an intracellular cytoplasmic kinase domain to transduce primary signals. LRR-RLKs have been reported to be involved in various plant processes, such as the development of reproductive organs and vegetative tissues, cell differentiation, and disease resistance in plants (Shiu and Bleecker, 2001; Wu et al., 2015). So far, hundreds of RLKs have been identiﬁed in many plant species, yet only few have been functionally characterized. In recent years, several central pathways of LRR-RLK-mediated signaling transduction have been identified in *Arabidopsis*. The BRI1ASSOCIATED KINASE-1 (BAK1) receptor, identified as a signaling partner of another LRR-receptor kinase, the brassinosteroid (BR) receptor BRI1 (BRASSINOSTEROID INSENSITIVE1), positively regulated a BR-dependent plant growth pathway (Li et al., 2002; Clouse, 2011). The BAK1, also named SERK3 (SOMATIC EMBRYOGENESIS RECEPTOR KINASE 3), belongs to the SERK subfamily, which consists of five homologs (SERK1–SERK5) (Aan den Toorn et al., 2015) . Since 2002, when BAK1 was first elucidated, several independent research groups have discovered BAK1/SERK3 and its homologs redundantly co-participated in a variety of biological processes, including BR signaling, light responses, root development, stomatal patterning, plant innate immunity, and floral organ abscission (Li et al., 2002; Heese et al., 2007; Chinchilla et al., 2009; Li, 2010; Meng et al., 2015; Li et al., 2018; Li et al., 2019). ERECTA (ER), another LRR receptor-like Ser/Thr kinase, functions synergistically with its two homologs ER-LIKE1 (ERL1) and ERL2 (forming the ER family) to regulate multiple developmental processes, including petiole length, inflorescence architecture, ovule development, and stomatal patterning (Torii et al., 1996; Shpak et al., 2004; Pillitteri et al., 2007a; van Zanten et al., 2009; Shpak, 2013).

It was reported that BRI1 KINASE INHIBITOR1 (BKI1) regulates plant architecture through coordinated inhibition of the BR and ER signaling pathways in *Arabidopsis* (Wang et al., 2017). BR early signaling, where BRI1 activation promotes the ER signaling by releasing the BKI1-mediated ER inhibition, enhances ER signaling cascades. SERK family members SERK1–SERK4 can regulate stomatal patterning by forming heterodimers with ER and its co-regulator TOO MANY MOUTHS (TMM) proteins upon ligand binding. Furthermore, SERKs perform functions downstream of the EPIDERMAL PATTERNING FACTOR (EPF) family (EPF1 and EPF2) and upstream of YODA (YDA) in regulating stomatal development (Shpak, 2013; Meng et al., 2015). The Mitogen Activated Protein Kinase (MAPK or MPK) cascade as well as MPKK4/MPKK5, MPK3/MPK6, and YODA were also used to transmit signals by ER for inflorescence architecture controlling (Meng et al., 2012; Shpak, 2013). These results indicate that the BR and ER signaling pathways can crosstalk at different levels and can, together, regulate multiple developmental processes (Meng et al., 2015; Wang et al., 2017).

Previously, a regulatory hotspot for *ZmBAK1-7*, identified by an eQTL analysis based on the RNA-seq data from 368 maize inbred lines, was shown to be associated with maize kernel development (Liu et al., 2015). The *ZmBAK1-7*, which we renamed *ZmRLK7* in this study, was predicted to encode a new LRR-RLK. Here, we try to understand the possible function of *ZmRLK7* through overexpression of either sense or antisense *ZmRLK7* sequence in *Arabidopsis*. Phenotypic analysis showed that sense *ZmRLK7* overexpression reduced the plant height, silique, seed, and petal size by regulating both cell expansion and cell proliferation, while the transgene of antisense *ZmRLK7* exhibited opposite phenotypes. Abnormal stomatal development was also observed in the sense *ZmRLK7* transgenic lines. Gene expression and genetic analysis results indicated that ZmRLK7 regulates transcript levels of BR, ER, and stomatal related genes and acts antagonistically with BAK1 to regulate plant architecture and organ size control.

## Materials and Methods

### Protein Sequence and Phylogenetic Analysis

To understand the relationship between ZmRLK7 and other homologous proteins, a multiple sequence analysis was performed on 17 protein sequences that were either annotated as LRR-RLKs in the literature or were identified in database searches by using a ZmRLK7 amino acid sequence as a criterion. A phylogenetic tree was constructed by using the neighbor-joining method with MEGA7.The protein structure of ZmRLK7 was predicted by searching against the InterPro database (http://www.ebi.ac.uk/interpro/) or SMART software (http://smart.embl-heidelberg.de/).

### Vector Construction and Plant Transformation

The full-length cDNA of *ZmRLK7* (GRMZM2G149051) was amplified from B73 cDNA libraries of young ears with gene-specific primers (**Supplementary Table S1**). The product was inserted between the maize *ubiquitin1* (*UBQ1*) gene promoter and nopaline synthase (Nos) gene terminator in the modified pCAMBIA3300 vector with *bar* as the selection marker gene at the restriction site *Kpn*I. After confirmation by sequencing, constructs with sense or antisense *ZmRLK7* (hereafter RS or RA) were introduced into either Col or *bak1-3* mutant plants using the *Agrobacterium tumefaciens* GV3101 strain for transformation.

### Plant Materials and Growth Conditions

The maize inbred line B73 (*Zea mays ssp. mays* var. *B73*) was grown in a greenhouse at conditions of 28°C day/25°C night, a 12 h light/12 h dark cycle, and relative humidity of 60% – 65%. Tissues of primary root, leaf, pre-pollinated cob, silk, husk, tassel, anthers, embryos, and endosperms of 13, 15, 20, 23, and 25 days after pollination (DAP) were frozen in liquid nitrogen immediately and stored at -80°C before RNA extraction.

The *Arabidopsis thaliana* ecotype Columbia (Col) was used as the wild type (WT). The *Arabidopsis* mutant *bak1-3* (*SALK_034523*) in the Col background was ordered from the Salk Institute (http://signal.salk.edu/cgi-bin/tdnaexpress/). WT and *bak1-3* were used to generate the transgenic lines overexpressing sense or antisense *ZmRLK7* followed the floral dip method (Clough and Bent, 1998). Seeds were surface-sterilized in 1% sodium hypochlorite and washed five times with sterilized water, and the transgenic lines were selected on MS media plates containing 10 mg/L glufosinate ammonium and verbalization at 4°C for 3 days before transfer to the phytotron (14 h light/10 h dark, 22°C day/20°C night, and 70% relative humidity). Positive seedlings and WT were transplanted into soil in growth chamber.

### Molecular Characterization of the Transgenic Lines and Mutant Plants

Leaf tissues from WT, *bak1-3*, and the transgenic lines were extracted for genomic DNA. Polymerase chain reaction (PCR) was used for verification of the presence of transgenes or T-DNA insertion. Primers used for detecting transgenic *Arabidopsis* were combinations of sense- or antisense-*ZmRLK7-*specific forward, respectively, with Nos reverse primers. Specific primers used to identify T-DNA insertion of *bak1-3* were obtained from the website (http://signal.salk.edu/tdnaprimers.2.html). Two combinations of primers were used for *bak1-3* mutant verification. PCR products were about 995 bp (from LP to RP) for WT and 520 bp (from LBb1.3 to RP) for homozygous lines. Mutant plants with homozygous T-DNA insertion were used in the experiment. The PCR parameters were 94°C for 5 min, 32 cycles of 94°C for 30 s, 55°C for 30 s, 72°C for 45 s, and a final 7 min at 72°C. All primer sequences are listed in **Supplementary Table S1**.

### Subcellular Localization

Full-length coding sequence of ZmRLK7 without stop codon was inserted upstream of the synthetic green fluorescent protein (sGFP), driven by the CaMV35S promoter. The resulting constructs expressing the ZmRLK7-GFP fusion protein were transformed into maize leaf protoplasts by a polyethylene glycol (PEG)/CaCl_2_-mediated transformation method (Wang et al., 2019). Fluorescent signals were examined and photographed by a laser scanning confocal microscope (Leica DMi8, Germany).

### Morphological and Cytological Analysis

The agronomic traits as plant heights, branch numbers were determined after pollination, and 1000-seed weight was determined when the plant matured, and each determination was repeated at least five times. Siliques, flowers, and seeds were scanned with a stereo microscope (Zeiss SteREO Discovery. V12, Germany) to produce digital images. For measuring cell size and cell number, petals were cleared in hydrate chloral/glycerol/H2O solution (8:2:1 w/v/v) (Yin et al., 2012) before being observed with a microscope (Nikon ECLIPSE Ni-U, Japan). The length of siliques, the length and width of petals and dry seeds, the cell areas, and cell numbers along the longitudinal and transverse axis of petals were calculated using Image J software. Each value represents measurements of 10 repeats except for cell areas of petals, each value of which represents measurements of more than 30 cells.

For epidermal cells and stomatal observations, the sixth fully expanded leaves were collected from 30-day-old plants. The abaxial leaf epidermis of WT and transgenic lines were separated, soaked in water for 4 h, and then cleared for 4 h in Hoyer’s solution containing 8:1:2 (w/v/v) hydrate/glycerol/water. All samples were observed and photographed using a Leica confocal microscope (DMi8, Germany). Numbers of pavement cells, stomata were counted in two square areas of 0.2 mm^2^ per leaf from 10 independent leaves. Only stomata with pores were counted. Stomatal index was calculated as percentage of the number of stomata forms to the total number of epidermal cells.

### Quantitative Real-Time PCR

Total RNA of tissues of maize and *Arabidopsis* were extracted using TRIZOL reagent (Invitrogen, USA) and PrimScript RT reagent Kit with gDNA eraser was used for cDNA reverse transcription (TAKARA, Japan). Approximately 1 µg of total RNA was used for DNaseI treatment and cDNA synthesis, and quantitative real-time PCR (qRT-PCR) analysis was performed with an ABI 7500 real-time PCR system (Applied Biosystems, USA) using SYBR Premix Ex Taq II (TaKaRa, Japan) and gene-specific primers. *AtUBQ10* was used as an internal control for *Arabidopsis*, and *ZmFPGS* was used for normalization of maize RNAs. Each qPCR assay was replicated for three biological replicates, and each biological replicate was examined in triplicate (Wang et al., 2019). The sequences of the primers are shown in **Supplementary Table S1**. The *Arabidopsis* mixed tissues of inflorescence (including florets and pedicels) and capsules of 0–7 DAP were used for RNA extraction and qRT-PCR assay.

### Statistical Analysis

Values are expressed as mean ± standard deviation (SD) relative to the wild-type data. The significant differences between values were analyzed by one-way ANOVA using Microsoft Excel 2019 and SPSS 26 (IBM, USA) program. LSD *post hoc* multiple comparison tests were performed and *p* ≤ 0.05 was considered as significant.

## Results

### Sequence Analysis and Phylogenetic Analysis of ZmRLK7

**Figure 1:**
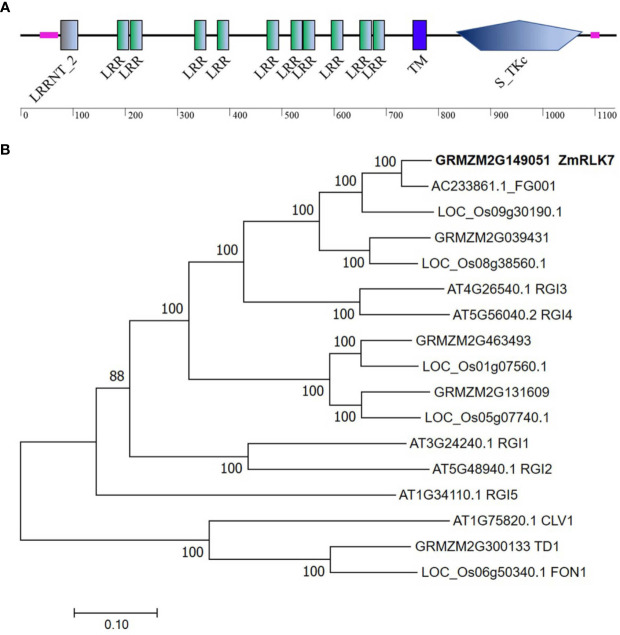
Protein structure and phylogenetic tree construction of ZmRLK7. **(A)** Protein structure prediction of ZmRLK7. The 10 tandem copies of leucine rich repeat (LRR), transmembrane (TM) and serine/threonine protein kinase (S_TKc) domain were denoted. **(B)** Amino acid sequences of ZmRLK7 and its homologs were aligned with ClustalW, and the phylogenetic tree was constructed using the neighbor-joining method in MEGA7. The 17 protein sequences used were either annotated in the literature or identified in database searches using ZmRLK7 amino acid sequence. The numbers at the nodes represented the credible values that were calculated from 1000-times bootstrap tests. Branch-length scale bars represent 0.20 amino acid substitutions per site.

The cDNA sequence of *ZmRLK7* (GRMZM2G149051) was isolated from B73 inbred line. The complete coding region of *ZmRLK7* was 3417 bp and predicted to encode a protein containing 1138 amino acids. Analyses of the structural properties of the *ZmRLK7*-predicted protein using the SMART program indicate that this protein is a putative leucine-rich repeat receptor-like kinase (LRR-RLK) featured with typical domains (**Figure 1A**). A putative extracellular domain (amino acids 195–699) in the N-terminal region, containing 10 tandem copies of a 24~25 amino acid leucine-rich repeat (LRR), and a transmembrane domain was identified at amino acid positions 755–777. In the C-terminal cytoplasmic region (amino acids 812–1092), a serine/threonine protein kinase domain was predicted.

To identify homologous proteins of ZmRLK7, a phylogenetic tree was constructed using the deduced protein sequences from various plant species (**Figure 1B**). There were five homologous genes in maize, and the closest homolog to ZmRLK7 was AC233861.1_FG001, which exhibits 92% identity. Sequence comparison revealed that the protein AT4G26450, namely Root Meristem Growth Factors Insensitive 3 (RGI3), has the highest similarity to ZmRLK7 in *Arabidopsis*. Five RGI members have been discovered in *Arabidopsis*, and they play redundant roles in perceiving RGF1 signals and controlling root meristem development (Ou et al., 2016). Sequence alignment indicated that there are 60% similarity in the amino acid sequences between ZmRLK7 and RGI3, however, the nucleotide sequence similarity is only about 40% (**Figure 1B** and **Supplementary Figure S1**).

### Expression Profiling and Subcellular Localization

**Figure 2:**
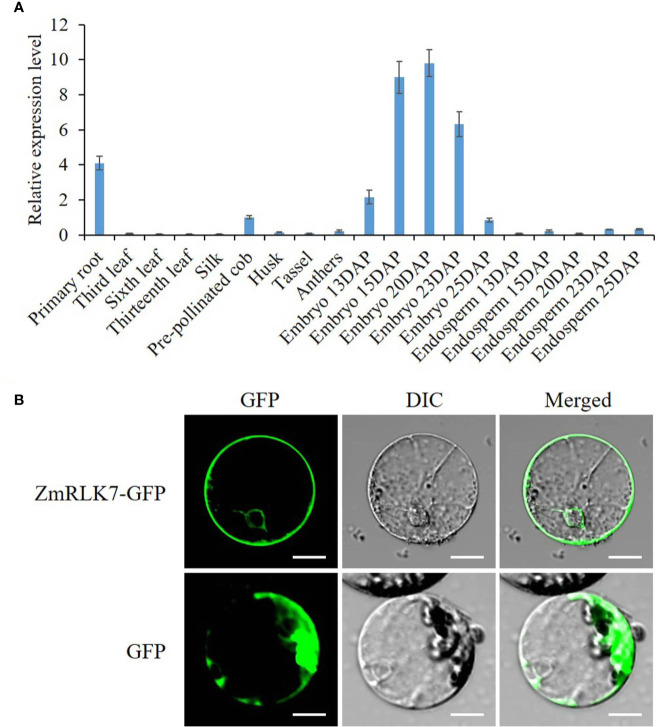
Transcriptional analysis and subcellular localization. **(A)** Tissue-specific expression analysis of *ZmRLK7* gene by qRT-PCR. Maize *ZmFPGS* mRNA level was used as internal control, and expression level of *ZmRLK7* in the pre-pollinated cob was used as a calibration. Values are means of three biological replicates with standard deviation (SD). DAP: days after pollination. **(B)** The ZmRLK7 protein were localization to both the plasma and nuclear membranes. GFP and the fusion protein ZmRLK7-GFP under the control of the CaMV35S promoter were transiently expressed in maize mesophyll protoplasts. DIC means differential interference contrast. Bars = 10 µm.

To determine expression pattern of *ZmRLK7*, qRT-PCR was performed on maize tissues of embryonic primary roots (3d after germination), leaves during vegetative growth, silk, pre-pollinated cob, husk, tassel, anthers, embryos, and endosperms of 13, 15, 20, 23, and 25 DAP. Results indicated that ZmRLK7 transcript was mainly detected in embryonic primary roots, embryos of 15–23 DAP, the pre-pollinated cob (**Figure 2A**). The expression level of *ZmRLK7* in embryos increased from 13 to 20 DAP, with the highest level in embryos of 20 DAP, and then decreased. These results suggested that *ZmRLK7* may play important roles in maize kernel development, especially the embryo development.

The subcellular localization of the ZmRLK7 protein is strongly associated with its biological function. We fused the sGFP to the C-terminus of ZmRLK7 and transiently expressed in maize mesophyll protoplasts. The green fluorescence signals indicated that ZmRLK7 protein was localized to both the plasma and nuclear membranes (**Figure 2B**), which was consistent with the structure prediction (**Figure 1A**).

### ZmRLK7 Affects Organ Size in *Arabidopsis*

**Figure 3:**
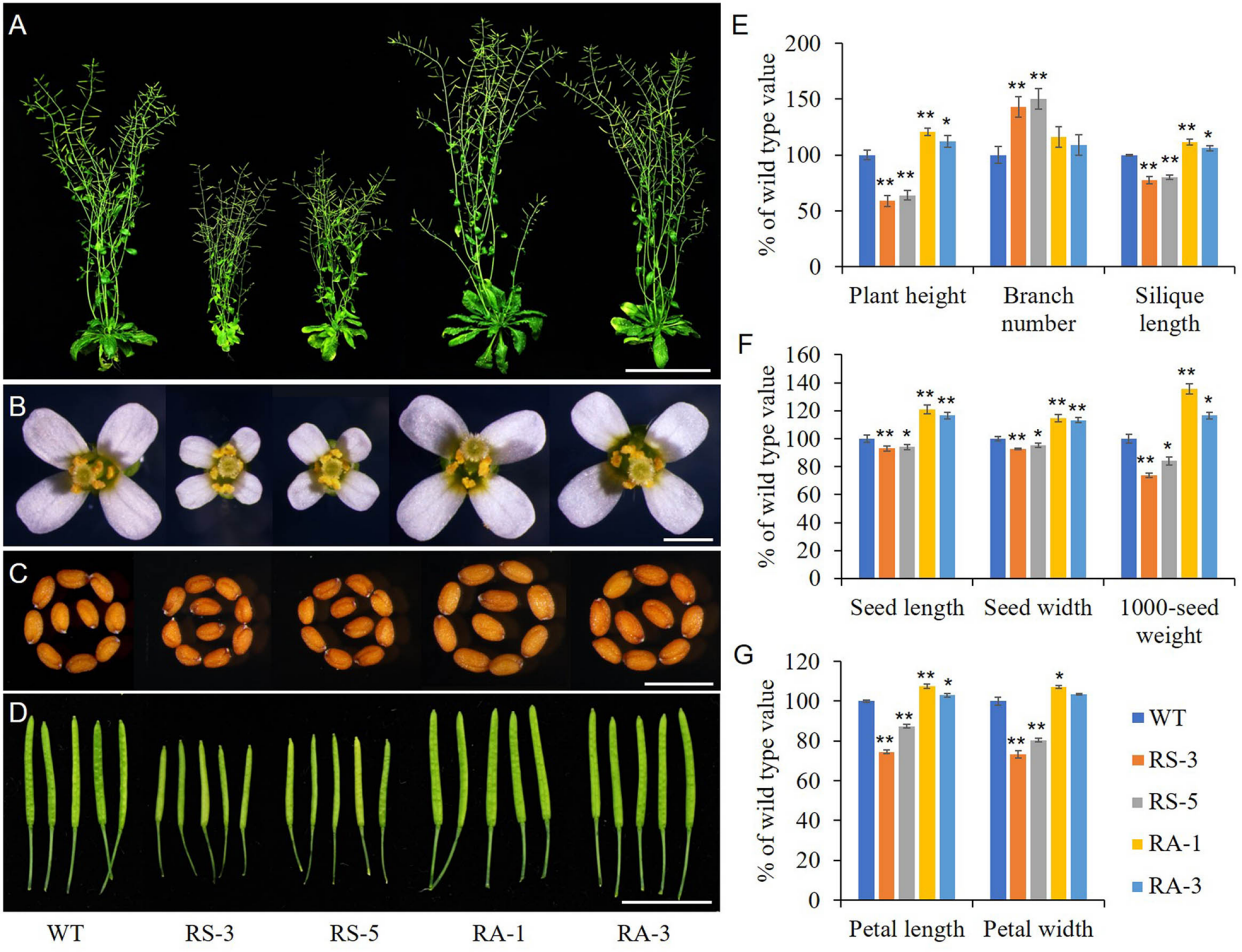
Morphological analysis of the homozygous transgenic lines and wild type (WT). **(A)** Whole morphology. **(B)** Flowers. **(C)** Seeds. **(D)** Siliques. **(E)** Statistical analysis of plant height, branch number and silique length. **(F)** Statistical analysis of seed length, seed width and 1000-seed weight. **(G)** Statistical analysis of petal length and width. WT, wild type; RS-3 and RS-5, *ZmRLK7* sense transgenic lines; RA-1 and RA-3, *ZmRLK7* antisense transgenic lines. Values are means with SD relative to the respective wild-type values. * and ** denote significant differences at *p* < 0.05 and 0.01 by ANOVA, respectively. Bars = 10 cm for **(A)**, 0.5 mm for **(B, C)** and 1 cm for **(D)**.

Transgenic *Arabidopsis* lines expressed sense or antisense *ZmRLK7* (hereafter RS or RA) in the Columbia background were used to evaluate the biological functions of *ZmRLK7*. Two representative events of RS or RA transgenic lines were selected for the following experiments (**Supplementary Figure S2A, B**). RS-3 and RS-5 were lines overexpressing sense *ZmRLK7*, and RA-1, RA-3 were lines overexpressing antisense *ZmRLK7*. The RS lines displayed a *bak1-3* mutant-like phenotype, including dwarf and smaller plant size compared with the WT, while the RA lines showed higher plant height and larger organs size both at the seedling and maturation stage (**Figure 3** and **Supplementary Figure 3A**). The flowering time of the RS lines was about 7–10 days earlier than that of WT, in contrast, the RA lines was later (2–3 days) compared to WT (**Supplementary Figure 3B**). Although RS-1 and RS-3 possessed more branches than that of WT and RA lines, the plant heights of RS-1 and RS-3 were only about 59% and 64% of WT, respectively (**Figures 3A** and **3E**). In contrast, the plant heights of RA-1 and RA-3 were significantly increased by 21% and 12%, respectively. When compared to WT, the flowers and seeds size of RS lines were smaller, while those of RA lines were larger (**Figures 3B**, **C**). In the RS lines, silique length, seed length, seed width and 1000-seed weight were significantly decreased, while they were significantly increased in the RA lines (**Figures 3D–F**). Seeds of the RA lines were 17–21% longer, 13–15% wider, and 17–35% heavier than that of WT (**Figures 3E**, **F**). Petal length and width of RS lines were also significantly smaller than those of WT, whereas petal length of RA lines and petal width of RA-1 were slightly bigger than WT (**Figures 3B**, **G**).

### ZmRLK7 Restrains Both Cell Expansion and Cell Proliferation in *Arabidopsis*

**Figure 4:**
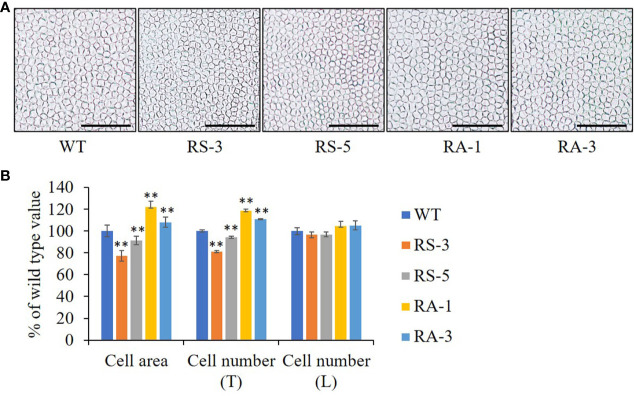
The petal cells size and numbers of different lines. **(A)** Adaxial petal cells in WT, the RS and RA transgenic lines. **(B)** Statistical analysis of cell areas and cell numbers along the transverse (T) and longitudinal (T) axis of petals. WT, wild type; RS-3 and RS-5, *ZmRLK7* sense transgenic lines; RA-1 and RA-3, *ZmRLK7* antisense transgenic lines. Values are means with SD relative to the respective wild-type values. Bars = 100 μm. ^**^ denote significant differences at *p* < 0.01 by ANOVA.

As the final size and shape of organs are determined by cell number and size formed (Mizukami, 2001), the petals of *Arabidopsis* which have a simple laminar structure were used to analyze organ growth and development (Xu and Li, 2011). The cell size and numbers were also measured on adaxial epidermis of petals. The epidermal cell size in the maximal width of petals was dramatically decreased (by 9–23%) in the RS lines, while they were significantly increased (by 8–22%) in the RA lines (**Figures 4A, B**). The numbers of adaxial epidermal cells in the petal-width direction (Transverse, T) was significantly decreased in the RS lines while increased in the RA lines when compared with the WT (**Figure 4B**). However, the number of adaxial epidermal cells in the petal-length direction (Longitudinal, L) was comparable in the RS lines, RA lines and WT, suggesting that ZmRLK7 restrain cell proliferation in the petal-width direction. These analyses indicated that overexpression of sense *ZmRLK7* restrains both cell expansion and cell proliferation in *Arabidopsis*, while the expression of antisense *ZmRLK7* enhances these traits.

### ZmRLK7 Affects the Stomata Development in *Arabidopsis*

Stomata are epidermal pores surrounded by two guard cells (GCs) that regulate gas and water vapor exchange, and stomatal patterning is critical for efficient regulation of gas exchange (Dow et al., 2014). In *Arabidopsis*, the stomatal lineage is initiated from a subset of protodermal cells that called meristemoid mother cells (MMCs). MMCs divide asymmetrically to create a small triangular cell, called a meristemoid (M), and a larger stomatal lineage ground cell (SLGC). Both cells may proceed with additional asymmetric cell division, and the meristemoid eventually differentiates into a round-shaped guard mother cell (GMC), which further divides symmetrically once into two GCs,and SLGC differentiates into pavement cells (Pillitteri and Torii, 2012). The placement and number of stomata in the epidermis are partly determined by the number of entries, amplifying and spacing divisions, which were accurately regulated by cell-cell signaling (Lau and Bergmann, 2012; Pillitteri and Torii, 2012; Pillitteri and Dong, 2013; Cheung and Wu, 2015). To determine whether ZmRLK7 affects stomatal development as the ERf or SERKs do, stomata number and structure were examined on leaf abaxial epidermis of the transgenic lines and WT. As shown in **Supplementary Figure S4A–4E**, there were abnormal epidemic cell structure in the RS lines, and the stomata number decreased obviously when compared to the WT and RA lines. As a result, the stomatal index of the RS lines was significantly lower than that of WT and RA lines (**Supplementary Figure S4F**). However, the RA lines showed no significant difference in both pavement cells structure and stomatal index compared with WT (**Supplementary Figure 4A–4F**). It seems that the divisions of meristemoids to GMCs or the entry of symmetric divisions from GMCs into GCs was disturbed in the RA lines, suggesting ZmRLK7 was also involved in regulating stomata development.

### ZmRLK7 Modulates Gene Expression Related to BR Signaling and ER Signaling Pathway

**Figure 5:**
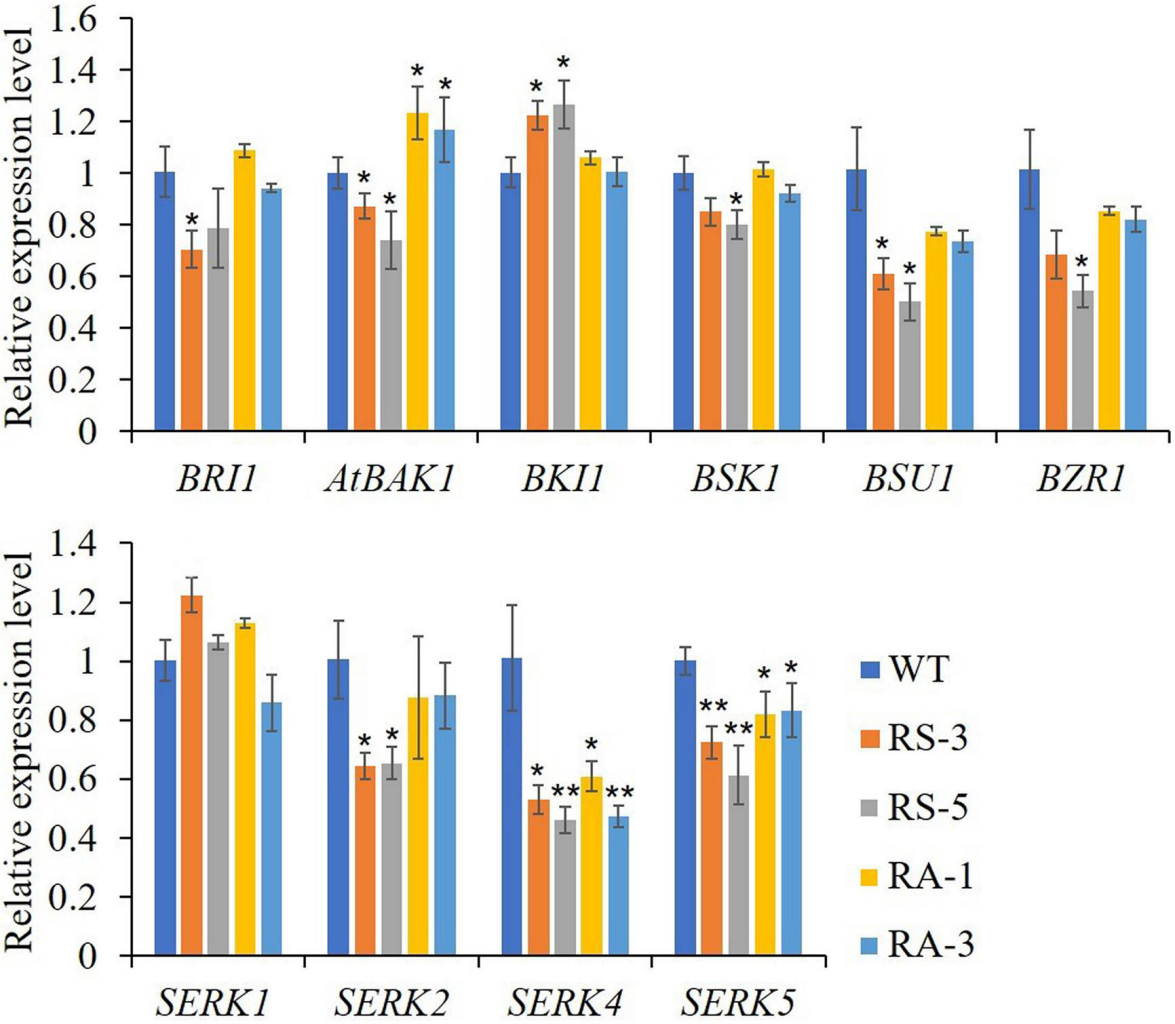
qRT-PCR analysis of genes related to BR signaling pathway and the SERKs family. WT, wild type; RS-3 and RS-5, *ZmRLK7* sense transgenic lines; RA-1 and RA-3, *ZmRLK7* antisense transgenic lines. *AtUBQ10* mRNA level was used as internal control. Values are means of three biological replicates with SD. ^*^ and ^**^ denote significant differences at *p* < 0.05 and 0.01 by ANOVA, respectively. All comparisons were with the WT as the control.

As previously reported, there were cross talks between SERKs, BR and ER signaling in regulating plant growth and stomatal development (Meng et al., 2015; Wang et al., 2017). In this study, we first selected genes that are involved in the BR signaling cascade. As shown in **Figure 5**, when compared with WT, the expression levels of *BRI1*, *BAK1*/*SERK3*, *BSK1*, *BSU1* and *BZR1*, which were positively involved in BR signaling pathway, were downregulated in the RS lines, while the negative regulator *BKI1* was significantly upregulated. In the RA lines, only the expression levels of *BAK1*/*SERK3* was significantly upregulated, the expression levels of the others had no significant alteration (**Figure 5A**). In RS lines, the expression levels of *SERK2*–*SERK5* were downregulated in RS lines, SERK4 dropping the most, while there were no significant changes for *SERK1* in either RS or RA lines. In the RA lines, *SERK4* and *SERK5* were significantly downregulated and *BAK1*/*SERK3* was upregulated significantly, while *SERK1* and *SERK2* showed no significant difference when compared to WT (**Figure 5B**). In order to investigate the role of ZmRLK7 in ER signaling pathway, we compared the expression levels of these genes previously reported to be involved in inflorescence architecture, ovule development, and stomatal formation and patterning (Shpak et al., 2004; van Zanten et al., 2009; Meng et al., 2012; Cai et al., 2017).

**Figure 6:**
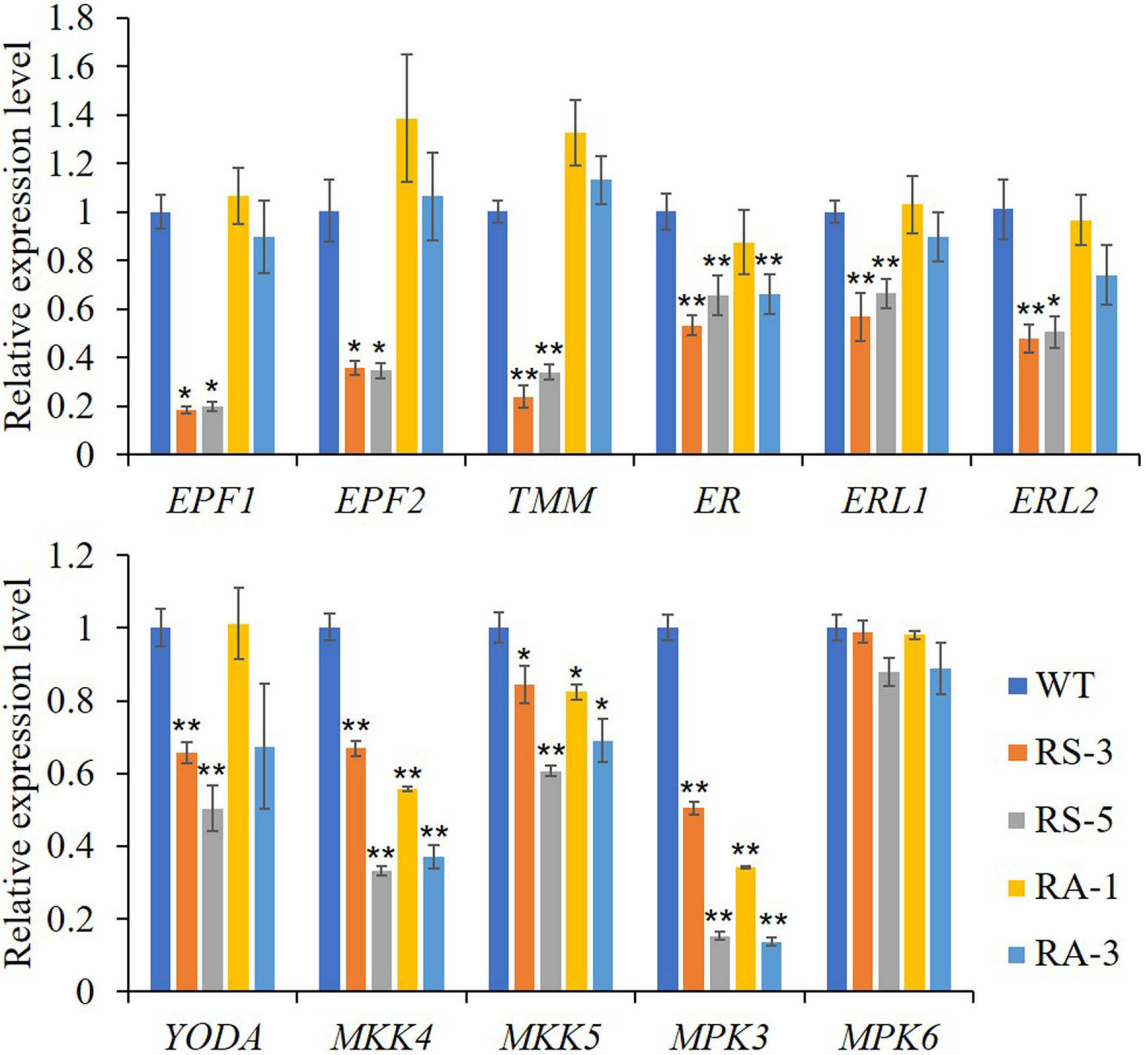
qRT-PCR analysis of genes related to ER signaling pathway. WT, wild type; RS-3 and RS-5, *ZmRLK7* sense transgenic lines; RA-1 and RA-3, *ZmRLK7* antisense transgenic lines. *AtUBQ10* mRNA level was used as internal control. Values are means of three biological replicates with SD. ^*^ and ^**^ denote significant differences at *p* < 0.05 and 0.01 by ANOVA, respectively. All comparisons were with the WT as the control.

As shown in **Figure 6**, the expression levels of ER and its partners TMM, ERL1, ERL2, EPF1, EPF2, and the downstream MAPK/MPK cascade, including YODA, MKK4/5, and MPK3, were dramatically downregulated in the RS lines compared with those in the WT, though with the exception of MPK6. Interestingly, MKK4/5 and MPK3 were dramatically downregulated in the RA lines like they were in RS lines, while other members had little changes in RA lines with the exceptions of TMM and EPF2 whose expression levels were upregulated compared to the WT. Three closely related basic helix-loop-helix (bHLH) transcription factors, SPEECHLESS (SPCH), MUTE and FAMA, are required for the transitions from MMC formation to GCs in the stomatal lineage (Ohashi-Ito and Bergmann, 2006; MacAlister et al., 2007; Pillitteri et al., 2007b; Pires and Dolan, 2010; Lau and Bergmann, 2012) were also determined. Consistent with the abnormal phenotypes in the RS lines epidermis, expression levels of the three genes were dramatically downregulated in the RS lines, especially for the *MUTE* gene (**Supplementary Figure S4G**). No significant differences between the RA lines and WT was found for the expression levels of *MUTE* and *FAMA*, however, the expression level of *SPCH* in RA plants was significantly increased. The above results indicated that ZmRLK7 affects inflorescence architecture, organ size, and stomata development by negatively regulating expression levels of genes involved in the BR and ER signaling pathway.

### Downregulation of *ZmRLK7* Substantially Rescues the *bak1-3* Mutant Phenotype

**Figure 7:**
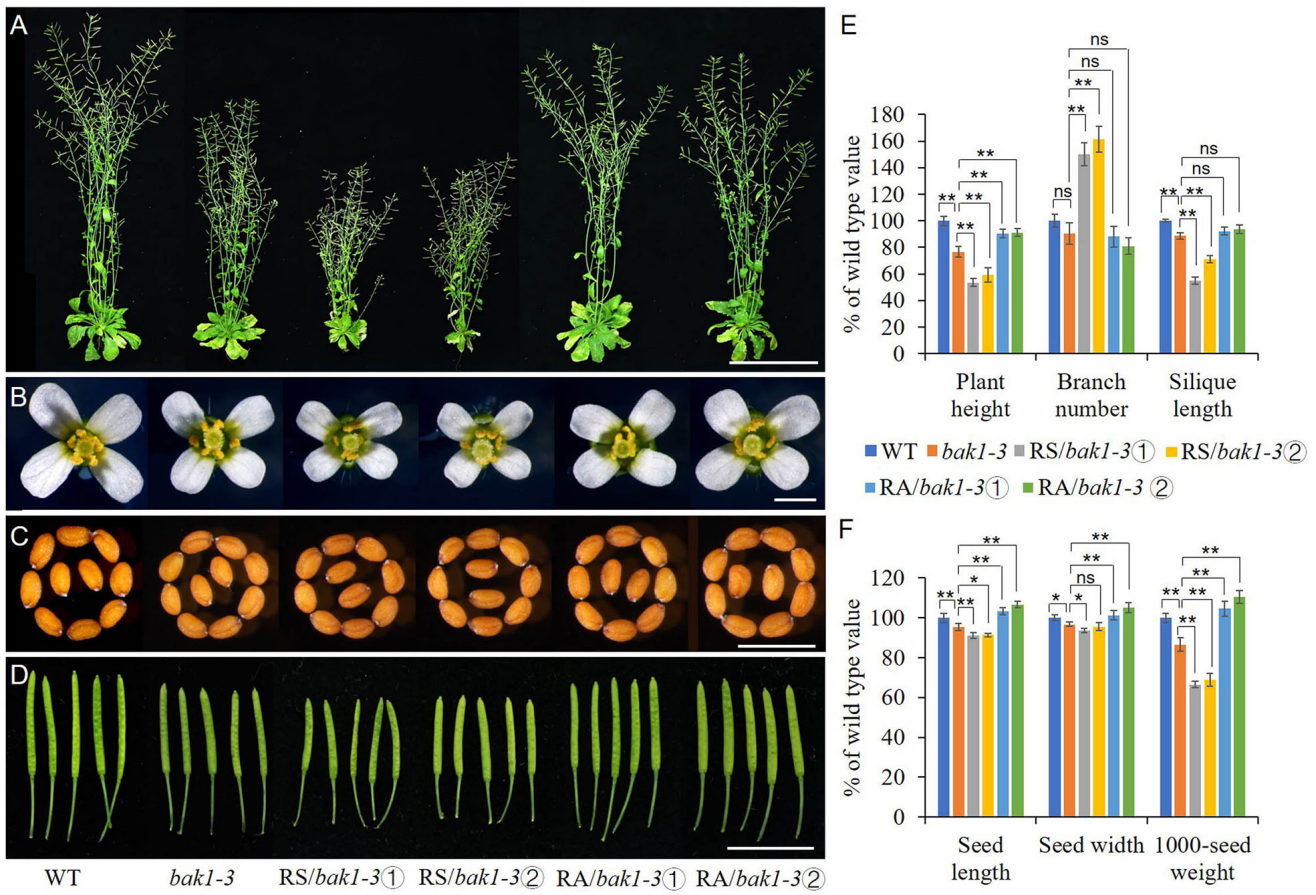
ZmRLK7 acted antagonistically with BAK1 on plant architecture, organ size and 1000-seed weight. **(A)** Whole morphology. **(B)** Florets. **(C)** Seeds. **(D)** Siliques. **(E)** Statistical analysis of plant height, branch number and silique lengths. **(F)** Statistical analysis of seed length, seed width and 1000-seed weight. WT, wild type; RS-3 and RS-5, *ZmRLK7* sense transgenic lines; RA-1 and RA-3, *ZmRLK7* antisense transgenic lines. Values are means with SD relative to the respective wild-type values. ^*^, ^**^ and ns denote significant differences at *p* < 0.05, 0.01 and no significant difference by ANOVA, respectively. Bars = 10 cm for **(A)**, 1 cm for **(B, D)** and 0.5 mm for **(C)**, respectively.

Due to the similar phenotype between RS lines and *Arabidopsis* loss-of-function mutant *bak1-3* and the opposite expression level of BAK1 in the RS and RA transgenic lines when compared to the WT, we expressed sense or antisense *ZmRLK7* in the *bak1-3* mutant background respectively. *bak1-3*, which has a T-DNA insertion in *AtBAK1*/*SERK3* gene (Chinchilla et al., 2007), was verified by PCR analysis and the homozygous were used for transformation (**Supplementary Figure S2C, D**). Two representative sense and antisense *ZmRLK7* transgenic *bak1-3* lines were selected respectively for further analysis. Compared with *bak1-3*, the RS/*bak1-3* lines showed more severe and additional phenotypes such as reduced plant height, reduced organ (flowers, seeds, and siliques) size, early flowering and increased branch number (**Figures 7A–D** and **Supplementary Figure S5**). In contrast, RA/*bak1-3* exhibited opposite phenotypes. Statistical analysis showed that the plant height of RS/*bak1-3* lines were significantly lower than that of *bak1-3* (by 23–30%) and WT (by 41–46%), while the RA/*bak1-3* lines were significantly higher, almost reaching the level of WT (90–91% of WT). As for RS lines, the number of branches of RS/*bak1-3* lines increased significantly (by 66–82%) compared with *bak1-3*, while there were no significant differences among RA/*bak1-3*, *bak1-3* and WT. Moreover, the seed length, seed width and 1000-seed weight of the RA/*bak1-3* lines were all significantly increased compared with *bak1-3* and there were no significant differences between RA/*bak1-3* and WT (**Figure 7F**). These results suggested that overexpression of antisense *ZmRLK7* could substantially rescue the *bak1-3* mutant phenotype to the wild-type level, while overexpression of sense *ZmRLK7* can make the mutant phenotype more severe. Combined with these results, we hypothesized that ZmRLK7 may function downstream of BAK1, and they had antagonistic effects on regulating plant development and organ size.

## Discussion

The growth and development of multicellular organisms is an important yet unanswered question in developmental biology. Since the first maize RLK gene (*ZmPK1*) was isolated (Walker and Zhang, 1990), more and more RLK genes with diverse functions have been identified (Li and Chory, 1997; Li et al., 2002). In this study, we have cloned a typical LRR-RLK gene *ZmRLK7* from B73 inbred line (**Figure 1**). Transient expression of ZmRLK7 in maize protoplasts results in plasma and nuclear membranes location (**Figure 2B**). The overexpression of antisense *ZmRLK7* in *Arabidopsis* (RA) resulted in more robust plants, enlarged organs (such as ovules, petals, siliques) and seeds size when compared with the wild type, while the sense *ZmRLK7* transgenic lines (RS) showed an opposite phenotype (**Figures 3** and **4**). The RA transgenic lines had the highest 1000-seed weight among the three lines while the RS lines had the lowest (**Figure 3F**). Furthermore, a lower stomatal index was observed in the RS transgenic lines, while it was not affected in the other two lines (**Supplementary Figure 4**).

Members of SERK family are essential regulators of BR perception and signaling *via* complexing with the BR receptor BRI1 (Li et al., 2002; Nam and Li, 2002; Karlova et al., 2006; Gou et al., 2012). SERK1, known to be involved in embryogenesis, also heterodimerizes with BRI1 and enhances BR signaling (Hecht et al., 2001; Karlova et al., 2006). The *bak1* null allele *bak1-3* mutant exhibits a weak *bri1-5* phenotype, and the overexpression of *BAK1* can suppress the weak *bri1-5*, and SERK4 was able to partially rescue *bri1-5* when overexpressed (Li et al., 2002; Nam and Li, 2002; He et al., 2007). SERK4, alternatively named BAK1-LIKE (BKK1), positively regulates a BR-dependent plant growth pathway and negatively regulates cell-death pathway in a BR-independent manner (He et al., 2007). In our RS transgenic lines, the expression of BAK1 was downregulated significantly while the BAK1 was significantly upregulated in the RA lines (**Figure 5A**). Besides BAK1/SERK3, the expressions of SERK2–SERK5 were significantly downregulated in the RS transgenic lines. Further, the RS/*bak1-3* lines were observed as having enhanced the *bak1-3* phenotype with additional phenotypes such as early flowering and increased branch number (**Figures 7A–D** and **Supplementary Figure S5**). When antisense *ZmRLK7* was overexpressed in the background of *bak1-3*, the plant height was almost recovered to the level of WT, the seed size and 1000-seed weight in RA/*bak1-3* lines increased significantly when compared with *bak1-3* and WT (**Figure 7**). These results may suggest *ZmRLK7* could regulate the plant growth and organs size through acting with the SERK family members, especially SERK3/BAK1.

The ER receptor, like BAK1, performs multiple functions in the *Arabidopsis* developmental processes and immunity. ER interacted with its two closely related LRR-RLK paralogs (ERL1 and ERL2) and the receptor-like protein TMM, synergistically regulated inflorescence architecture, lateral organ shape, ovule development, stomatal patterning, and transpiration efficiency (Torii et al., 1996; Shpak et al., 2003; Shpak et al., 2004; Shpak et al., 2005). In this study, the expression levels of ER and its partners TMM, ERL1, ERL2, EPF1, EPF2, and the downstream MAPK cascade, including YODA, MPKK4/5, and MPK3, were dramatically downregulated in the RS lines compared with WT. Interestingly, only MPKK4/5 and MPK3 were downregulated in the RA lines like in RS lines, while other members had little changes in RA lines. Moreover, the expression level of MPK6 had little changes in all lines (**Figure 6**). Too many studies have shown that SPCH acts as the point of signal integration to regulate stomatal patterning. The protein stability of SPCH is modulated by positional clues composed of ligands, EPF1 and EPF2 peptides, and receptors, including the ER family (ER, ERL1, and ERL2) and the TMM (Pillitteri et al., 2007b; Shpak, 2013). In this study, the overexpression of sense *ZmRLK7* induced the abnormal stomata development and resulted in a lower stomatal index, and the expression levels of SPCH, MUTE, and FAMA FAMA almost decreased to 20% of they were in the WT (**Supplementary Figure 4**). These results suggested that ER signaling cascade was the main component that most affected by overexpression of *ZmRLK7*. BR signaling cascade was always concerned in the regulating of plant development (Clough and Bent, 1998; Gou et al., 2012). The BRI1 was activated in the present of BR on the membrane and then heterodimerized with BAK1. The two vital transcription factors BRI1 EMS SUPPRESSOR1 (BES1) and BRASSINAZOLE RESISTANT1 (BZR1) were sequentially changed their status through phosphorylation to transmit signals downstream (Li et al., 2018). SERKs interact with BRI1 to positively regulate BR response in a redundancy manner (Li et al., 2002; Gou et al., 2012). In this study, the expression levels of genes related to the BR cascade were examined. As shown in **Figure 5**, compared with the WT, transcription of all these genes were downregulated in the RS lines, with similar results of SERKs and ER cascade. These suggested that *ZmRLK7* synergistically regulates plant development and stomatal patterning by influencing the SERKs, BR cascade, and ER cascade.

BKI1 was a negative regulator of BRs and the ER signaling pathway (Jiang et al., 2015). The expression level of BKI1 maintained no obvious changes in RA lines, while it was significantly higher in RS lines compared with it was in the WT (**Figure 5A**), which indicating that ZmRLK7 plays a BKI1-like negative regulator and acts as a bridge between BRs and ER signaling pathway. Including the BAK1 and ER-associated developmental processes such as plant height, silique, seeds, and stomatal patterning, we also found that the overexpression of sense *ZmRLK7* in *Arabidopsis* increased the branch numbers in RS lines compared with that in the RA, WT, and *bak1-3* plants (**Figures 3E** and **7E**). Besides, overexpression of sense *ZmRLK7* in the *bak1-3* mutant and Col background both promoted flowering time (**Supplementary Figure S3B** and **S5**). Furthermore, the homolog of ZmRLK7 in *Arabidopsis* RGI3, belongs to RGIs family which play redundant roles in controlling root meristem development. The other four members can be detected in the root tips, except for RGI3 (Ou et al., 2016), indicating that RGI3 may possess unidentified functions. In this study, the RGI3 expression level was also determined, and it increased significantly both in RS lines and RA-1 line when compared to that in the WT (**Supplementary Figure S5A**), which was interesting and unexpected. This may be due to the low nucleotide sequence identity between *ZmRLK7 *and *RGI3*, and the functional redundancy of RGIs family, which makes it difficult to be clearly predicted.

Together, these results in sense or antisense *ZmRLK7* overexpression in different backgrounds (WT and *bak1-3*), leading to changes in plant architecture and organ size as well as changes in gene expression levels involved in the SERK family, ER, and BR signaling cascade; it is suggested that *ZmRLK7* is important in controlling plant architecture and organ size formation across different species. This work provides a better understanding of the biological function of *ZmRLK7*. Further study in maize is underway.

## Data Availability Statement

All datasets presented in this study are included in the article/**Supplementary Material**.

## Author Contributions

CH and LW designed this study. JW, CH, HG, CL, QL, TL, and RD performed the experiment. JW, CH, and HG analyzed the data. CH and JW wrote the manuscript. All authors contributed to the article and approved the submitted version.

## Funding

This work was supported by the Major Project of China on New varieties of GMO Cultivation (2016ZX08003003-006), Natural Science Foundation of Shandong Province (ZR2017MC040), and National Natural Science Foundation of China (31971964).

## Conflict of Interest

The authors declare that the research was conducted in the absence of any commercial or financial relationships that could be construed as a potential conflict of interest. 

## Supplementary Material

Supplementary Figure 1 Alignment of the amino acid sequences. ZmRLK7 was aligned with its four maize homologs, *Arabidopsis* RGI3 (AT4G26540.1) and rice LOC_Os09g30190.1 using BioEdit software with default options. The 10 leucine-rich repeat (LRR), transmembrane and protein kinase domain are underlined. Amino acid positions are shown on the top, and identical and conserved residues are marked as asterisks and dots/colons, respectively.

Supplementary Figure 2 PCR verification of the transgenic lines and *bak1-3*. **(A)** Verification of *ZmRLK7* sense transgenic lines. **(B)** Verification of *ZmRLK7* antisense transgenic lines. **(C)** T-DNA insertion sites of *Arabidopsis* *bak1-3* mutant with exons shown as black boxes. **(D)** PCR verification of *bak1-3* mutant. M, DL2000 marker; RS-3 and RS-5, sense *ZmRLK7* transgenic lines; RA-1 and RA-3, antisense *ZmRLK7* transgenic lines; RS/*bak1-3*① and ② are sense *ZmRLK7* transgenic lines in the background of *bak1-3*; RA/*bak1-3*① and ② are antisense *ZmRLK7* transgenic lines in the background of *bak1-3*; WT, wild type; P, plasmid control.

Supplementary Figure 3 Morphology at vegetative stage. **(A)** and flowering stage **(B)**. WT, wild type; RS-3 and RS-5, sense *ZmRLK7* transgenic lines; RA-1 and RA-3, antisense *ZmRLK7* transgenic lines. Bars = 2 cm for (A) and 5 cm for (B).

Supplementary Figure 4 Stomata phenotypes at leaf abaxial epidermis. **(A–E)** stomatal index **(F)** and qRT-PCR analysis for *SPCH*, *MUTE* and *FAMA* **(G)** WT, wild type; RS-3 and RS-5, sense *ZmRLK7* transgenic lines; RA-1 and RA-3, antisense *ZmRLK7* transgenic lines. *AtUBQ10* gene was used as internal control. Values are means of three biological replicates with SD. * and ** denote significant differences at *p* < 0.05 and 0.01 by ANOVA, respectively. Bars = 100 μm. All comparisons were with the WT as the control.

Supplementary Figure 5 qRT-PCR analysis of *RGI3* **(A)** and flowering date of WT and *bak1-3* **(B)**. WT, wild type; RS-3 and RS-5, sense *ZmRLK7* transgenic lines; RA-1 and RA-3, antisense *ZmRLK7* transgenic lines; RS/*bak1-3*, sense *ZmRLK7* transgenic lines in the background of *bak1-3*. Bar = 5 cm.
