# Supplementary figures and images for: Overexpression of an Antisense RNA of Maize Receptor-Like Kinase Gene ZmRLK7 Enlarges the Organ and Seed Size of Transgenic Arabidopsis Plants
Source: Front Plant Sci. 2020 Nov 11;11:579120. doi: 10.3389/fpls.2020.579120 (PMC7693544; doi:10.3389/fpls.2020.579120)

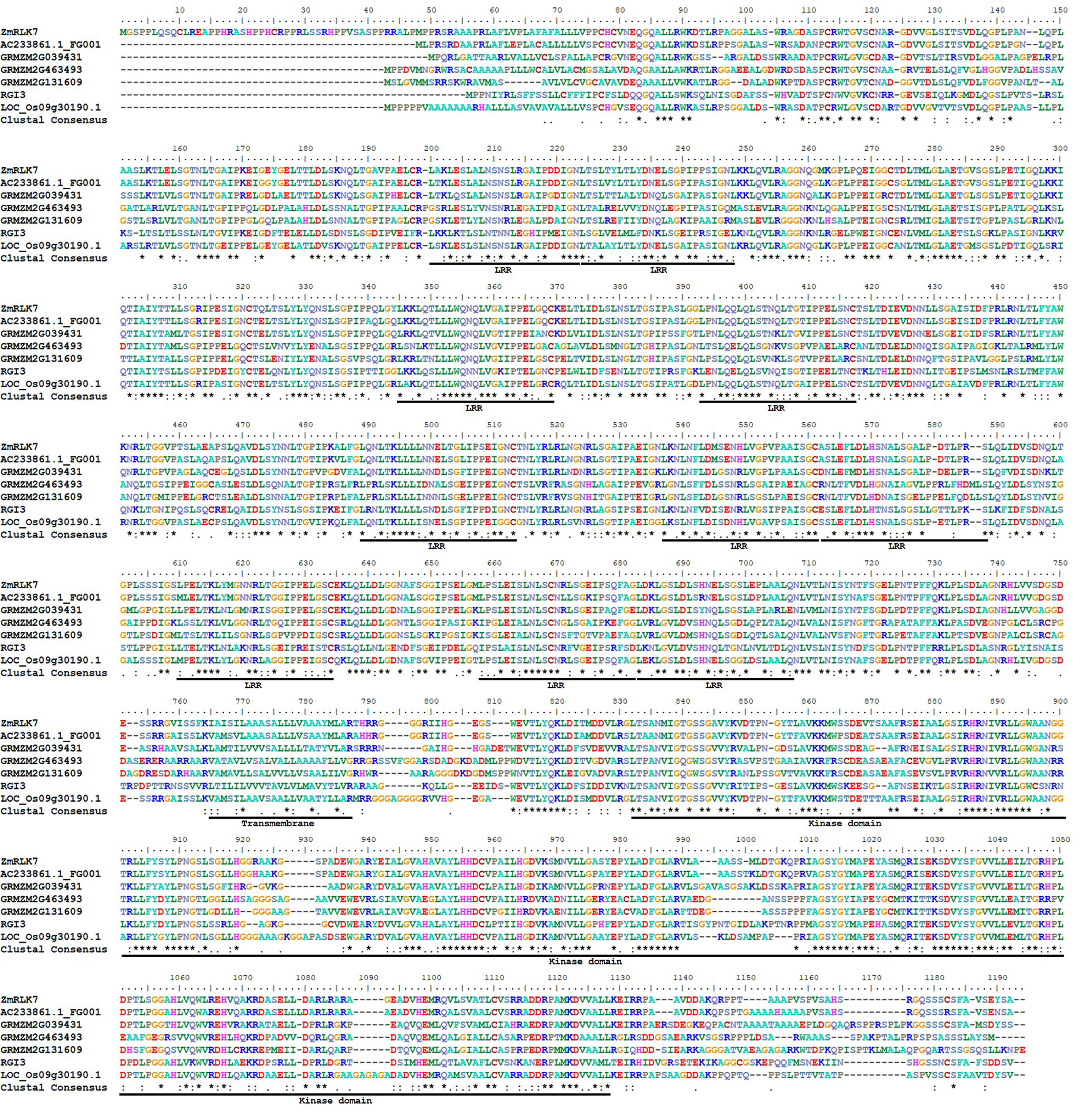

Supplement: Supplementary Figure 1 — Alignment of the amino acid sequences. ZmRLK7 was aligned with its four maize homologs, Arabidopsis RGI3 (AT4G26540.1) and rice LOC_Os09g30190.1 using BioEdit software with default options. The 10 leucine-rich repeat (LRR), transmembrane and protein kinase domain are underlined. Amino acid positions are shown on the top, and identical and conserved residues are marked as asterisks and dots/colons, respectively. [file Image_1.jpeg]

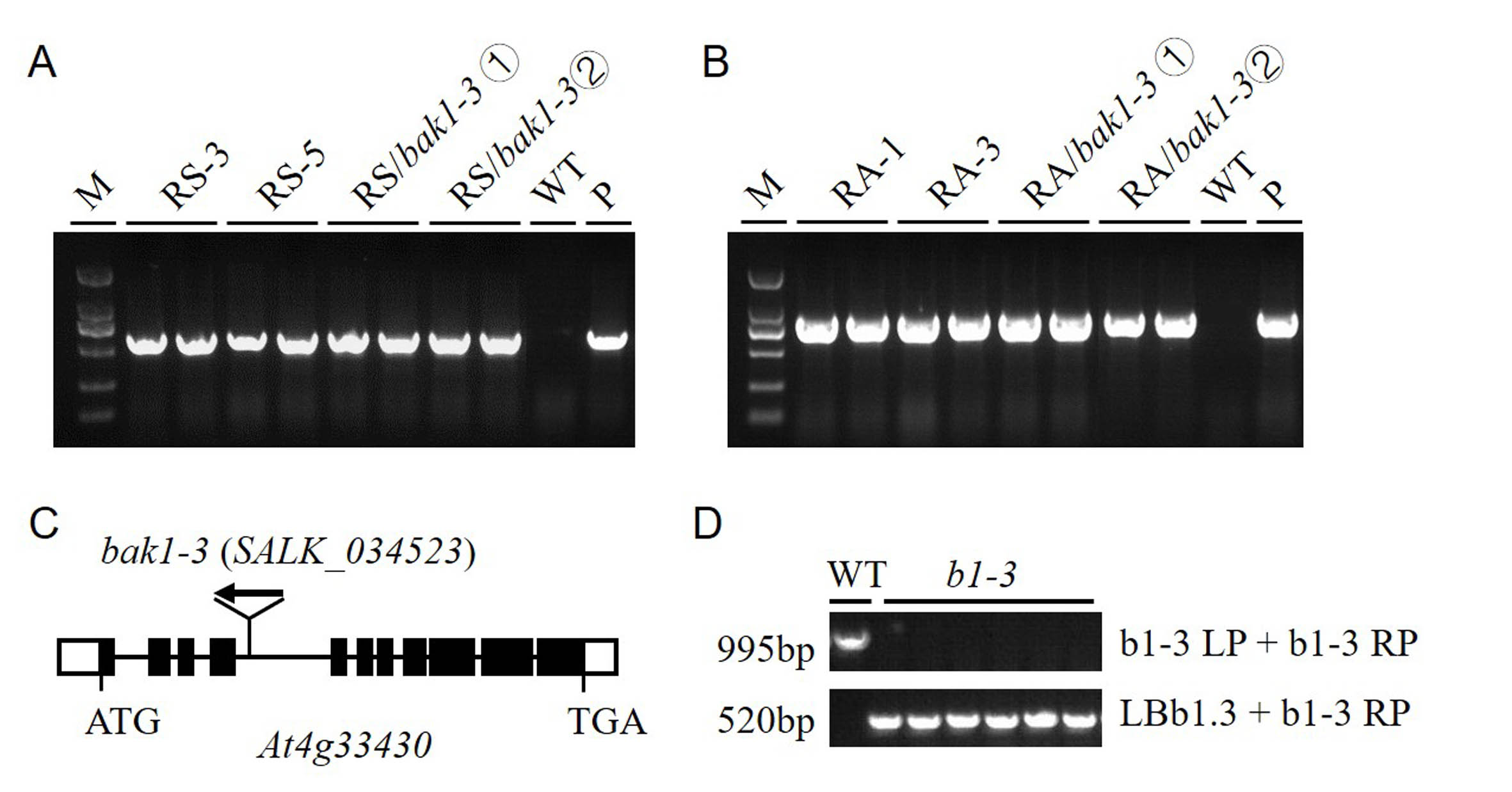

Supplement: Supplementary Figure 2 — PCR verification of the transgenic lines and bak1-3. (A) Verification of ZmRLK7 sense transgenic lines. (B) Verification of ZmRLK7 antisense transgenic lines. (C) T-DNA insertion sites of Arabidopsis bak1-3 mutant with exons shown as black boxes. (D) PCR verification of bak1-3 mutant. M, DL2000 marker; RS-3 and RS-5, sense ZmRLK7 transgenic lines; RA-1 and RA-3, antisense ZmRLK7 transgenic lines; RS/bak1-3① and ② are sense ZmRLK7 transgenic lines in the background of bak1-3; RA/bak1-3① and ② are antisense ZmRLK7 transgenic lines in the background of bak1-3; WT, wild type; P, plasmid control. [file Image_2.jpeg]

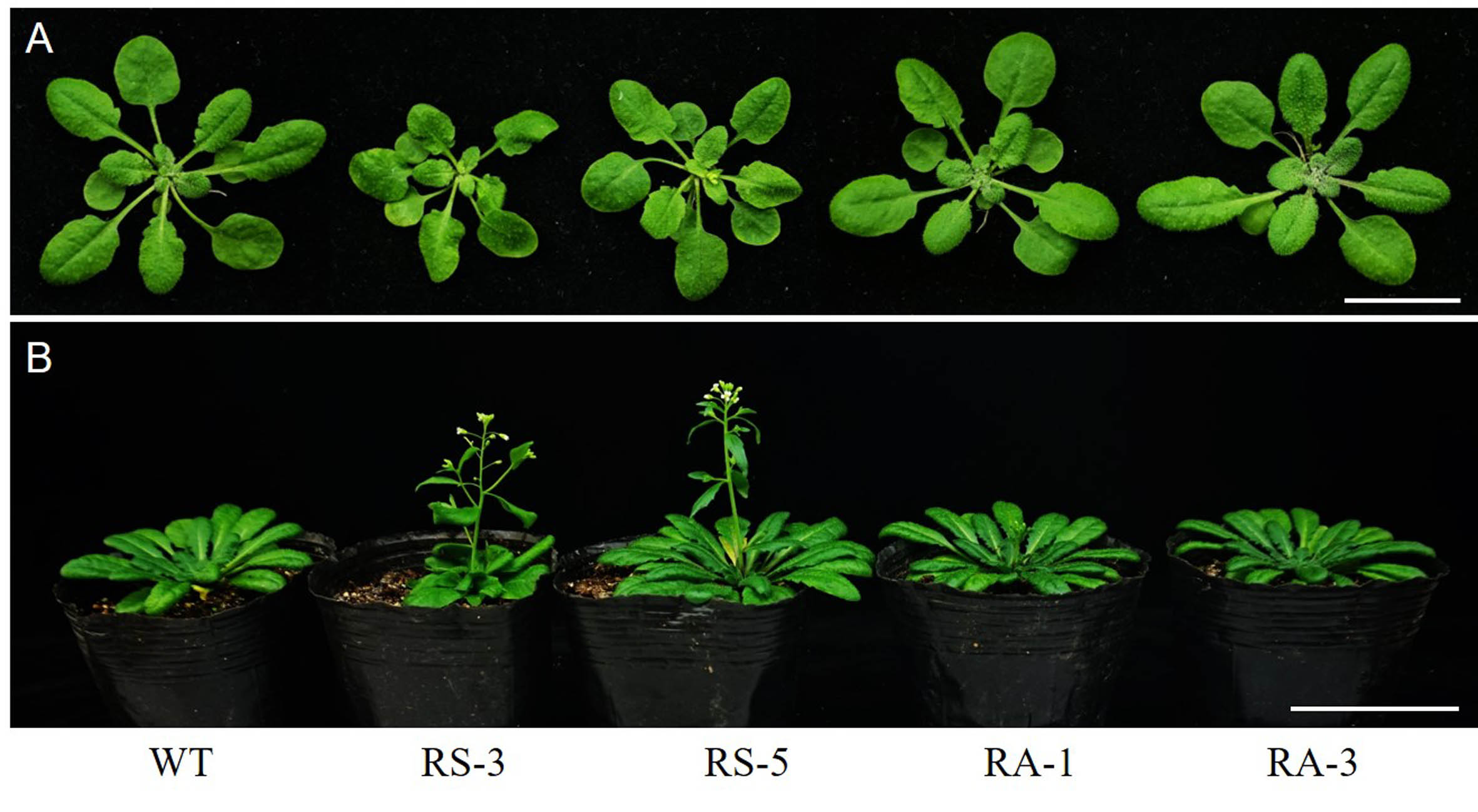

Supplement: Supplementary Figure 3 — Morphology at vegetative stage. (A) and flowering stage (B). WT, wild type; RS-3 and RS-5, sense ZmRLK7 transgenic lines; RA-1 and RA-3, antisense ZmRLK7 transgenic lines. Bars = 2 cm for (A) and 5 cm for (B). [file Image_3.jpeg]

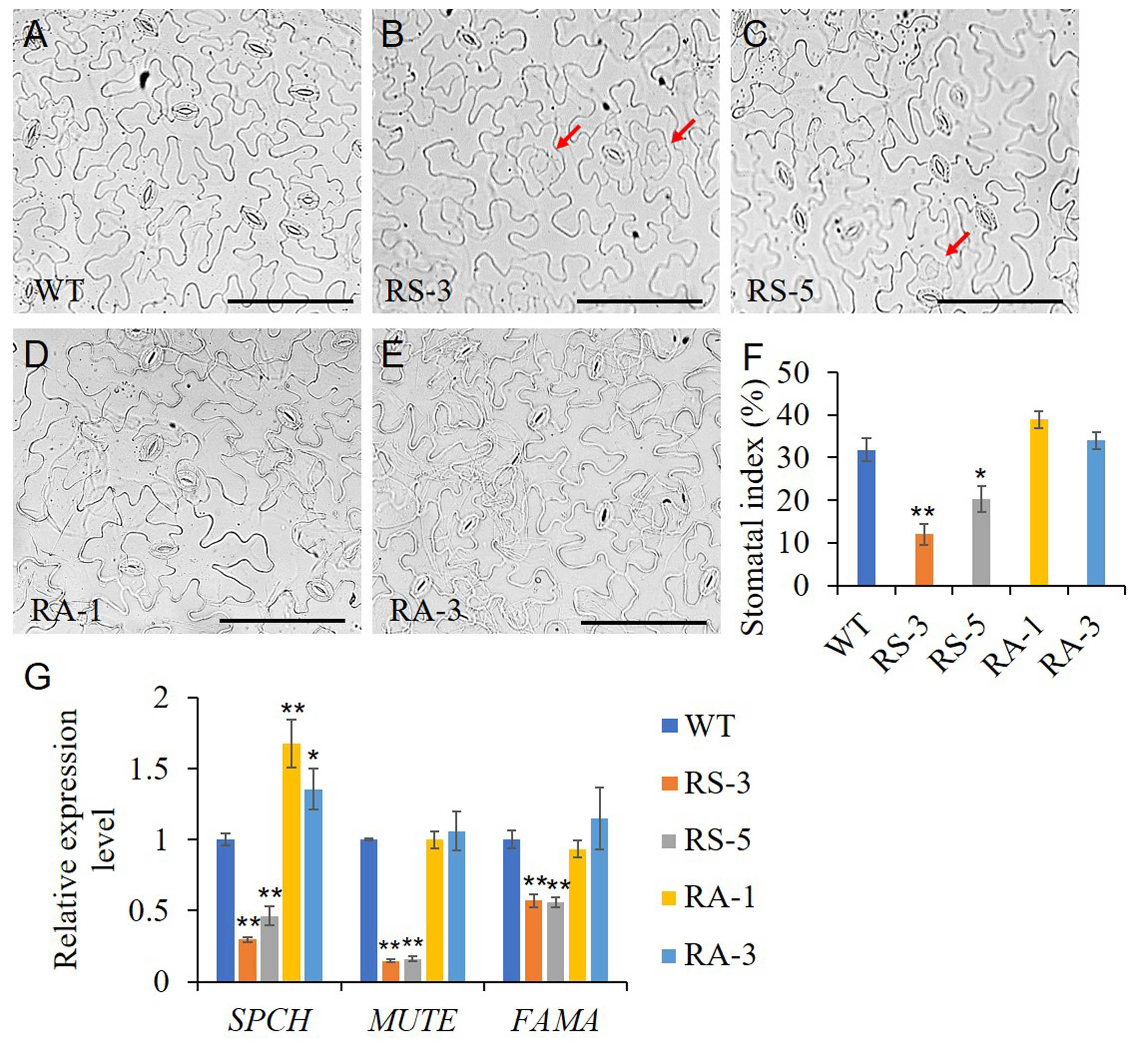

Supplement: Supplementary Figure 4 — Stomata phenotypes at leaf abaxial epidermis. (A–E) stomatal index (F) and qRT-PCR analysis for SPCH, MUTE and FAMA (G) WT, wild type; RS-3 and RS-5, sense ZmRLK7 transgenic lines; RA-1 and RA-3, antisense ZmRLK7 transgenic lines. AtUBQ10 gene was used as internal control. Values are means of three biological replicates with SD. * and ** denote significant differences at p < 0.05 and 0.01 by ANOVA, respectively. Bars = 100 μm. All comparisons were with the WT as the control. [file Image_4.jpeg]

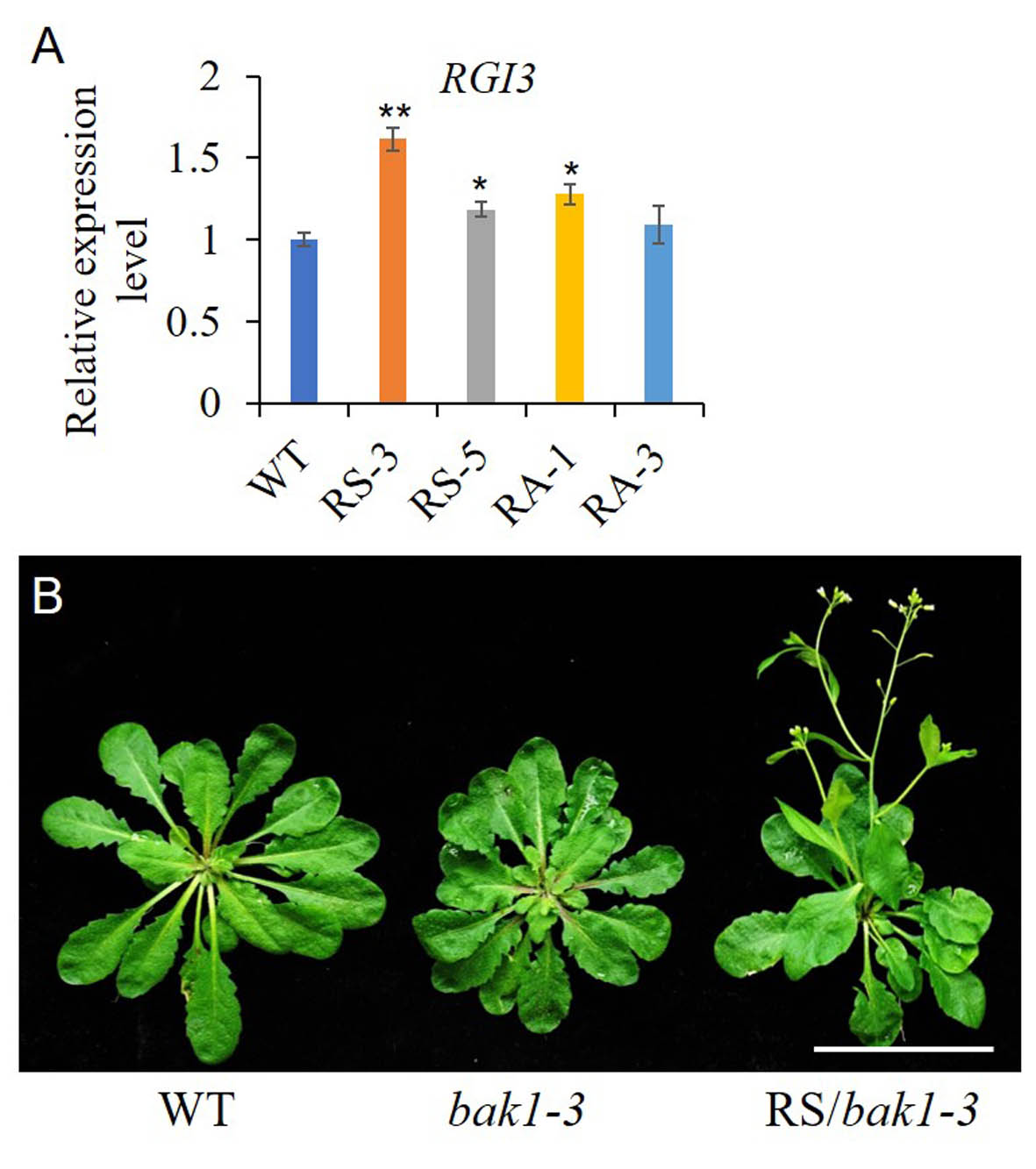

Supplement: Supplementary Figure 5 — qRT-PCR analysis of RGI3 (A) and flowering date of WT and bak1-3 (B). WT, wild type; RS-3 and RS-5, sense ZmRLK7 transgenic lines; RA-1 and RA-3, antisense ZmRLK7 transgenic lines; RS/bak1-3, sense ZmRLK7 transgenic lines in the background of bak1-3. Bar = 5 cm. [file Image_5.jpeg]
