# Supplementary material for: Overexpression of an Antisense RNA of Maize Receptor-Like Kinase Gene ZmRLK7 Enlarges the Organ and Seed Size of Transgenic Arabidopsis Plants
Source: Front Plant Sci. 2020 Nov 11;11:579120. doi: 10.3389/fpls.2020.579120 (PMC7693544; doi:10.3389/fpls.2020.579120)
Supplement: Supplementary file 6 [file Table_1.docx]

**Table S1** Primers used for plasmid construction, PCR, RT-PCR, and qRT-PCR

| **Primer name** | **Primer sequence (5’ - 3’)** | **Description** |
| --- | --- | --- |
| **Primers used for plasmid construction** | | |
| RLK7-F | GGTACCGTAGTACAAACAATTCATGGGGTC | RA, RS |
| RLK7-R | GGTACCAAGGCTGCTACAAAAACCGACATT |  |
| GFP1-F | TCTAGAGGATCCATGGGGTCTCCCCCTCTA | ZmRLK7-GFP |
| GFP1-R | CCCTTGCTCACCATGGAGGCAGAGTACTCCGAC |  |
| **Primers used for PCR verification of transgenic plants** | | |
| RS-R | TCGCAAGCATTGGGATTGTAGAG | RS verification |
| AS-R | AAGCGTGACGCGTCCGAGCTAC | RA verification |
| NOS-R | GACTCTAATCATAAAAACCCATCTC |  |
| LBb1.3 | ATTTTGCCGATTTCGGAAC |  |
| b1-3 LP | GAAAAACAGTTTAGCCGACCC | *bak1-3* verification |
| b1-3 RP | CATTCTTCCAGAACCAAATCG |  |
| **Primer used for RT-PCR and qRT-PCR** | | |
| RLK7-QF | AGTCCATGAACGTGCTGCT | *ZmRLK7* (*GRMZM2G149051*) |
| RLK7-QR | TACTCTGGTGCCATGTAGCC |  |
| UBQ10-QF | CCTTGTATAATCCCTGATGA | *AtUBQ10* (*AT4G05320*) |
| UBQ10-QR | AACAGGAACGGAAACATAGT |  |
| FPGS-QF | ATCTCGTTGGGGATGTCTTG | *ZmFPGS* (*GRMZM2G393334*) |
| FPGS-QR | AGCACCGTTCAAATGTCTCC |  |
| BRI1-QF | TTTGACCCCGAGCTTATGAAG | *BRI1* (*At4g39400*) |
| BRI1-QR | CCCGGCTTGTATCTCCTTAAAC |  |
| BAK1-QF | CCGATGTCTTTGGGTATGGAG | *BAK1* (*AT4G33430*) |
| BAK1-QR | CAACCCTTTCACCCAGTCTAG |  |
| BSU1-QF | CGGCTTTGAAAGATTCGCTG | *BSU1* (AT1G03445) |
| BSU1-QR | GGTGAGGATGAATCAGCTTAGG |  |
| BZR1-QF | CTTTGTGTTGAAGCTGGTTGG | *BZR1* (*AT1G75080*) |
| BZR1-QR | GGTATTCCTTCATCGCTTCCTC |  |
| BSK1-QF | AGAGACCACTTTCGCCATTAG | *BSK1* (*AT4G35230*) |
| BSK1-QR | TGATAACTCGTTTGTGCCCTC |  |
| BKI1-QF | CATCTCCGACTAATAGTGGCC | *BKI1* (*AT5G42750*) |
| BKI1-QR | CGCTTGAGTTCTTGCAATGAG |  |
| SERK1-QF | AATCATTCACTGTGGCTTGCTTCTG | *SERK1* (*AT1G71830*) |
| SERK1-QR | GTGCAAGGATTCACTAGCGTAGGAT |  |
| SERK2-QF | TGCCTCTTCTAACATGGAAGGTGAT | *SERK2* (*AT1G34210*) |
| SERK2-QR | AGTACACGGATTAACAAGCGTAGGA |  |
| SERK4-QF | GAGTCGCTGGAAACGCTGAAGG | *SERK4* (*AT2G13790*) |
| SERK4-QR | TGGAGTACATTGTTTGCAGGGTCAC |  |
| SERK5-QF | CATAAGCGGTCCCATCCCTTCCT | *SERK5* (*AT2G13800*) |
| SERK5-QR | ATCCAGCGGCAGAGCAGTCA |  |
| EPF1-QF | TACCAACATCCTCCCATCCAAGTCA | *EPF1* (*AT2G20875*) |
| EPF1-QR | CGTGTGAGCAATCTGGCAACCT |  |
| EPF2-QF | ATTCGCACGCCGCCACTAA | *EPF2* (*AT1G34245*) |
| EPF2-QR | CCGGTAAGCTTGATCCTGTTGG |  |
| TMM-QF | CGGAGCTACGGACGTGTCAA | *TMM* (*AT1G80080*) |
| TMM-QR | ACTCGGGAAGTATTTACAGCAGTTT |  |
| ERACTA-QF | TTCTTGGGTTTCTCTTCTGCTTGAG | *ERACTA* (*AT2G26330*) |
| ERACRA-QR | CGAAGAAGGTGAAGTTGTCCAGTC |  |
| ERL1-QF | CGGCTATGAACAACGAAGGGAAAG | *ERL1* (*AT5G62230*) |
| ERL1-QR | AACACCTCGCCAAGAACACAAGT |  |
| ERL2-QF | AGGATAGAGACCATGAAAGGCTTGT | *ERL2* (*AT5G07180*) |
| ERL2-QR | TTCGCCACGTTGCTGAATGAAG |  |
| YODA-QF | AATGGGAACCGAACACCAGTAAACA | *YODA* (*AT1G63700*) |
| YODA-QR | CCGCCTAGGAGATGCAGACAGA |  |
| MKK4-QF | CTTCCGGTGGTTCCGGTGGC | *MKK4* (*AT1G51660*) |
| MKK4-QR | TCCACCTGCTCCGCTTCCGA |  |
| MKK5-QF | CCGGCTACGGCGTCTCAGGA | *MKK5* (*AT3G21220*) |
| MKK5-QR | CGGCGGCAACATTTGACGGA |  |
| MPK3-QF | CCGGCGGTGGAAACTCACGG | *MPK3* (*AT3G45640*) |
| MPK3-QR | CGTCTCCGTATCCAACACAGAGCA |  |
| MPK6-QF | GGACTCTCCGTGAGATCAAGCTGC | *MPK6* (*AT2G43790*) |
| MPK6-QR | AGCGTTTCTTAATGGTGGCGGGA |  |
| SPCH-QF | TTGCGTTCTCTTATGCCTTGTTTCT | *SPCH* (*AT5G53210*) |
| SPCH-QR | GCTTAGGACTTCGGCGTAGGTT |  |
| MUTE-QF | AGCATGGAGGAGACTGTCTTATACT | *MUTE* (*AT3G06120*) |
| MUTE-QR | TGGTAGAGACGATCACTTCATCAGA |  |
| FAMA-QF | GCTGCTTTGGAGGATCTTCATCTCT | *FAMA* (*AT3G24140*) |
| FAMA-QR | GGAACTTGCTATGTCTTCTGCCGTA |  |
| RGI3-QF | GGCTGGCTCCTATGGTTACATGGC | *RGI3* (*AT4G26540*) |
| RGI3-QR | TGTGCACCTCCTGGTAGATCCG |  |
